# Comparative genomics of *Bacillus thuringiensis *phage 0305φ8-36: defining patterns of descent in a novel ancient phage lineage

**DOI:** 10.1186/1743-422X-4-97

**Published:** 2007-10-05

**Authors:** Stephen C Hardies, Julie A Thomas, Philip Serwer

**Affiliations:** 1 Department of Biochemistry, University of Texas Health Science Center at San Antonio, 7703 Floyd Curl Drive, San Antonio, Texas 78229-3900, USA

## Abstract

**Background:**

The recently sequenced 218 kb genome of morphologically atypical *Bacillus thuringiensis *phage 0305φ8-36 exhibited only limited detectable homology to known bacteriophages. The only known relative of this phage is a string of phage-like genes called BtI1 in the chromosome of *B*. *thuringiensis israelensis*. The high degree of divergence and novelty of phage genomes pose challenges in how to describe the phage from its genomic sequences.

**Results:**

Phage 0305φ8-36 and BtI1 are estimated to have diverged 2.0 – 2.5 billion years ago. Positionally biased Blast searches aligned 30 homologous structure or morphogenesis genes between 0305φ8-36 and BtI1 that have maintained the same gene order. Functional clustering of the genes helped identify additional gene functions. A conserved long tape measure gene indicates that a long tail is an evolutionarily stable property of this phage lineage. An unusual form of the tail chaperonin system split to two genes was characterized, as was a hyperplastic homologue of the T4gp27 hub gene. Within this region some segments were best described as encoding a conservative array of structure domains fused with a variable component of exchangeable domains. Other segments were best described as multigene units engaged in modular horizontal exchange. The non-structure genes of 0305φ8-36 appear to include the remnants of two replicative systems leading to the hypothesis that the genome plan was created by fusion of two ancestral viruses. The case for a member of the RNAi RNA-directed RNA polymerase family residing in 0305φ8-36 was strengthened by extending the hidden Markov model of this family. Finally, it was noted that prospective transcriptional promoters were distributed in a gradient of small to large transcripts starting from a fixed end of the genome.

**Conclusion:**

Genomic organization at a level higher than individual gene sequence comparison can be analyzed to aid in understanding large phage genomes. Methods of analysis include 1) applying a time scale, 2) augmenting blast scores with positional information, 3) categorizing genomic rearrangements into one of several processes with characteristic rates and outcomes, and 4) correlating apparent transcript sizes with genomic position, gene content, and promoter motifs.

## Background

We have reported the DNA sequence and genomic annotation of a novel large genome bacteriophage named *Bacillus thuringiensis *phage 0305φ8-36 [1,2]. Phage 0305φ8-36 was isolated from soil while targeting the isolation of large, unusual phages of unsampled or undersampled types [3-6]. Examination of phage 0305φ8-36 by electron microscopy revealed an unusually long contractile tail, and three large corkscrew shaped fibers emanating from the upper aspect of the baseplate [4]. The genes of 0305φ8-36 have only distant homologues and the gene for the large terminase subunit was reported to be anciently derived [4]. Among the functionally annotated gene products [1,2] are a putative RNA polymerase, DNA polymerase III and associated replicative and metabolic enzymes, two DNA primases, and virion proteins. A thorough survey by mass spectrometry identified 55 virion protein-encoding genes, and noted that this was an excess over the prototypical myovirus, T4, and particularly so if tabulated in terms of the total length and hence complexity of virion protein sequence.

The closest homologues of most of the virion protein-encoding genes and a few replicative genes were found to reside in a single segment of the chromosome of *B. thuringiensis *serovar *israelensis*. A smaller segment also appears in the chromosome of a closely related species, *B. weihenstephanensis*. These two phage-like regions are termed BtI1 and BwK1, respectively [1]. In this report, a detailed study is made of the genomic organization and vertical descent of phage 0305φ8-36 in comparison with BtI1/BwK1.

A central problem in comparative genomics analysis is to reconcile the high incidence of horizontal exchanges [7-10] with the observation of conserved gene organization [11]. Some elements of gene order in the genes encoding virion proteins appear to have been conserved in many widely different types of tailed phages, despite these phages being anciently related [12]. The most commonly observed organization of phage genes, includes 1) a conserved order of genes within a head structure and morphogenesis module, and 2) a conserved order of modules for head, tail, baseplate, and tail fiber proteins [11]. This most frequent organization is not found in all phages. In particular, T4 encodes its virion proteins in several genomic segments interspersed with non-virion genes, although functional clustering persists within the segments [13]. The implications of gene order for annotating other large myoviral genomes has been discussed [14]. Phage 0305φ8-36 conforms to this relatively common gene organization in most respects, but it has novel genes implicated in curly fiber formation placed on both sides of the head structure module [1].

A relatively strong conservation of gene organization implies a relatively light load of horizontal transfers. Phage 0305φ8-36 lacks genes recently transferred from other known phage or bacterial genomes [1]. T4-like phages share this feature, and are therefore a useful model for analyzing 0305φ8-36. The T4 genome organization was found to be substantially conserved over a very long time [15,16]. This supports the proposition that obligatory lytic phages may be less prone to horizontal transfer and hence less prone to reorganization of their genome plan than are temperate phages [17,18]. An expectation of a particular gene order can be valuable in hypothesizing functional assignments for genes that have diverged beyond easy recognition. This becomes especially true now that there are more elaborate comparative methods to follow up on such a hypothesis. For example, we have demonstrated a strategy of using gene order in combination with weak Blast scores to propose a distant homology, and then following up with comparison of predicted secondary structures [19].

To positionally evaluate weak blast matches in a systematic way across the 0305φ8-36 genome, this study used a computational method that presents its results through the graphics display program Gbrowse [20]. This allowed definition of insertions and deletions (indels) relating 0305φ8-36 and BtI1/BwK1 down to the domain level, and a visual collation of the results with the distribution of other 0305φ8-36 features. One of the major sources of confusion in achieving a totally automated comparison of genomes was the incidence of paralogues. It was found to be most useful to find the paralogues first as part of the basic Psi-Blast searches for each gene and to represent them within the same graphics display as the chains of 0305φ8-36 versus BtI1/BwK1 Blast matches.

Using these and other comparative techniques, we found that between 0305φ8-36 and BtI1/BwK1 there was an extensive conservation of gene order among the virion protein-encoding genes. This was in spite of numerous large and small insertions or deletions interspersed with the conserved matches. The time over which this arrangement persisted was estimated to be 2 – 2.5 billion years (Byr). Within this conserved framework, several multigene modules encoding virion proteins have apparently inserted. The content of genes encoding virion proteins in these modules accounts for the greater complexity of virion proteins compared to other myoviruses, e.g. T4. Finally, an evolutionary scenario for the creation of the overall 0305φ8-36 genome plan is explored in which two ancestral phages are fused and then resolved to a single genome plan which still contains remnants of both replication systems.

## Results

### Phage 0305φ8-36 BtI1 comparison

#### Phage 0305φ8-36 gene organization suggests an origin from two major ancestors

**Figure 1:**
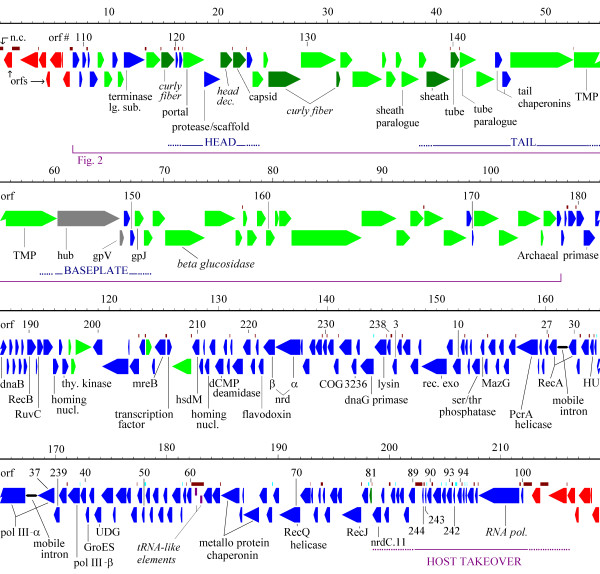
**Map of the genome of 0305φ8-36 showing distribution of features**. The features are from ref. [1]. The scale is in kilobase pairs. Arrows – orfs color coded as: green – encodes virion protein, dark green – encodes high copy virion protein, grey – implied virion protein by sequence analysis only, blue – non-structural, and red – non structural in terminal repeat. The orf number for every 10th orf is given, with the exception of numbers that are not consecutive, for which each orf is labelled. Purple rectangles – tRNA-like sequences of unclear significance. Abbreviations include: TMP – tape measure protein; thy. kinase – thymidine kinase; mreB – mreB-like rod determination protein; hsdM – HsdM, Type I restriction-modification system methyltransferase subunit; nrd – ribonucleoside reductase; rec. exo – DNA repair exonuclease; UDG – uracil-DNA glycosylase. Italic indicates a tentative assignment. Noncoding regions greater than 40 bp are marked above the orfs in cyan if they do, or brown if they do not, contain a promoter candidate of the class described in Figure 6.

The gene organization of phage 0305φ8-36 [1,2] is shown in Figure 1. The transcriptional orientation of most orfs converges on the center of the genome, dividing it into a left arm and a right arm. The left arm bears a relationship to a string of phage-like genes in a contig [GenBank:NZ_AAJM01000001] from the draft sequence of *B. thuringiensis israelensis*. This phage-like chromosomal region is called BtI1. BtI1 contains the closest known homologues for 1) many 0305φ8-36 structure and morphogenesis genes, and 2) four non-structure genes on the left arm (orf180, a primase, a helicase, and recB) [1]. The homology relationships of the right arm (discussed below) are completely unlike the left arm. The difference in relationships of the left and right arms combined with their opposite transcriptional orientations are the first of several indications that the 0305φ8-36 genome plan may have been created by the fusion of separate left and right arm ancestors.

The few virion protein-encoding genes dispersed in the right arm (orfs 205, 209, 81) have the appearance of morons – genes acquired relatively recently by single gene horizontal transfer and often transferred together with their own promoters and transcription terminators [8]. All three prospective morons are preceded by a non coding space suitable to carry a promoter. Orfs 209 and 81 are followed by a transcriptional terminator indicated by an obvious hairpin followed by an oligo T tract (not shown). Although orf 205 is not followed by a transcriptional terminator, it is inverted relative to the surrounding genes. Hence, all three are transcriptionally isolated from their neighbors, as expected for structure genes acquired by insertion into non structure modules after the generation of the initial genome plan. In contrast, the three virion protein-encoding genes at the right end of the left arm (orfs 197, 198, 199) are part of an apparent large polycistronic operon including the left arm non-structure genes. Hence, these are thought to have arrived in the initial fusion, and the boundary of the postulated fusion coincides with the major inversion junction. This implies a separate ancestry of the left and right arm non structure genes.

#### Phage 0305φ8-36 genes are only distantly related to known viral and cellular genes

To estimate the time to the common ancestor of the 0305φ8-36 left arm and BtI1, the divergence of its six most heavily conserved protein sequences was tabulated (Table 1). These were found comparable to the divergence of the same T4 genes between T4 and the exo T4-even phages P-SSM2 and S-PM2 [21]. The exo T-even phages are the most divergent members of the T4 superfamily, and were estimated to be 2.5 – 3.2 Byr diverged from T4 itself [15]. This estimate was based on recently improved divergence time estimates for their cyanobacterial host species from *E. coli *made by Battistuzze *et al*. [22]. It was argued that the phages were at least as divergent as their hosts because the phage DnaB, clamp loader, and RecA genes are more divergent than their host counterparts. Further support for an ancient split between 0305φ8-36 and BtI1 came from the global tree for the large subunit of the phage DNA packaging ATPase/terminase [3]. The upper splits on that tree correspond to host differences such as Gram negative versus Gram positive, or the proteobacterial diversification. Those splits are also in the 2.5 – 3.2 Bya range on the Battistuzzi *et al*. [22] time scale. The terminase divergences of those splits are about 75% (not shown). This would place the 0305φ8-36/BtI1 split in the 2.0 – 2.5 Bya range. Hence, 0305φ8-36 is just close enough to BtI1 to consider these as divergent members of the same superfamily. But 0305φ8-36 is at least 2.0 Byr diverged from BtI1, so they should not be considered close relatives. The even greater divergence of the 0305φ8-36 proteins from the nearest phage of an established viral type is also shown in Table 1. These numbers place 0305φ8-36/BtI1 outside of any established myoviral phage genus. Similarly, the 0305φ8-36 large terminase joined the global terminase tree at the root [4], consistent with an extremely ancient origin.

**Table 1:** Divergence of homologous proteins of 0305φ8-36 and BtI1 compared to divergence among T4-like phages

***Terminase (large subunit)***	**D (%)^1^**	***Portal***	**D (%)^1^**
0305φ8-36 vs. BtI1	69	0305φ8-36 vs. BtI1	57
0305φ8-36 vs. KPP95^2^	72	0305φ8-36 vs. HF1 gp94	77
T4 vs. P-SSM2	65	T4 vs. P-SSM2	62

***Capsid***		***Sheath***	

0305φ8-36 vs. BtI1	60	0305φ8-36 vs. BtI1	71
0305φ8-36 vs. b.p. 37 orf013	79	0305φ8-36 vs. HF2p095	79
T4 vs. P-SSM2	65	0305φ8-36 vs. KVP40	84
		T4 vs. S-PM2	63

***Helicase***		***Primase***	

0305φ8-36 vs. BtI1	58	0305φ8-36 vs. BtI1	69
0305φ8-36 vs. Nil2	76	0305φ8-36 vs. phBC6A51	73
T4 gp41^3 ^vs. P-SSM2	58	T4 gp41^3 ^vs. S-PM2	68

^1^Divergence is (100 – percent identity) from a Psi-Blast alignment. Divergence was not corrected for saturation.

^2^Phage hosts are as follows: HF1 – *Halobacterium*; KPP95 – *Klebsiella*; P-SSM2 -*Prochlorococcus *(a cyanobacterium); S-PM2 – *Synechococcus *(a cyanobacterium); Bacteriophage 37 – *Staphylococcus*; phBC6A51 – putative prophage of *Bacillus cereus; *Nil2 – prophage of *Escherichia coli*.

^3^Residues 179–382 of T4 gp41 were used for the helicase comparison, and 1–178 for the primase.

No second descendant of the proposed right arm ancestor is currently available for comparison. Only a few of the 0305φ8-36 right arm genes have genes of named phages as their closest homologue [1]. Other than homing nucleases, these include the MazG gene, and two paralogues, orf61 and 88, of unknown function each distantly matching genes in *B. cereus *phage phBC6A51. Ignoring genes with no detected homologues, most other right arm gene products match proteins from Gram positive bacteria, but only slightly better than they match proteins of Gram negative bacteria. The Gram positive/negative split is set at approximately 3.2 Bya on the Battistuzzi *et al*. [22] time scale. Hence, the right arm has also descended without substantial exchange of genes with known viral or bacterial lineages for approximately 3 Byr.

#### A comparative study of the virion protein-encoding genes between 0305φ8-36 and BtI1 reveals a detailed conservation of gene order

**Figure 2:**
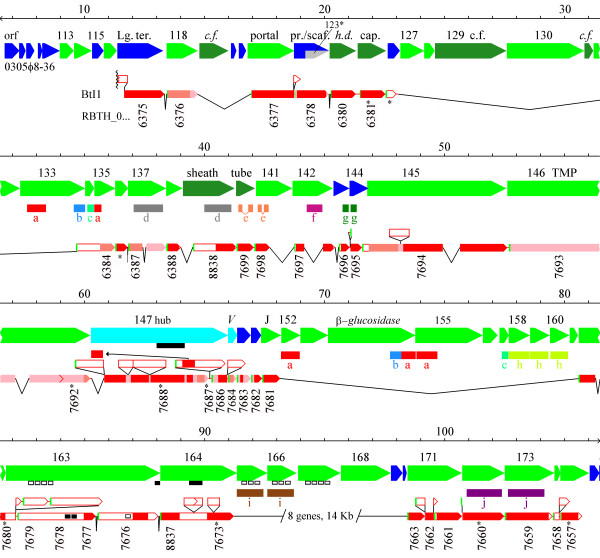
**Main structure-encoding region of 0305φ8-36 showing similarities to BtI1 and paralogous domains**. The figure was modified from Gbrowse output as described in the methods. Phage 0305φ8-36 orfs are color coded as in Figure 1. BtI1 orfs are color coded as follows: Green – N terminus of a BtI1 gene. Shades of red from bright to pale indicate assignment of homology with increasing reliance on positional information as described in the methods. The size of a connector dropping below the chain of matches indicates the amount of DNA missing in BtI1 versus 0305φ8-36. A triangle above the chain of matches indicates the amount of DNA in BtI1 in excess over 0305φ8-36. Boundaries of BtI1 frames marked with an asterisk were revised over those indicated in GenBank. Red angle brackets fuse two BtI1 orfs by correcting a frameshift. The left end of the BtI1 chain of glyphs is at the end of a contig. Colored rectangles below the 0305φ8-36 orfs indicate paralogous domains in 0305φ8-36. Open black boxes immediately under 0305φ8-36 orfs or within BtI1 orfs indicate FN3 domains. Closed black boxes indicate domains as follows: Under orf147 – T4gp27 domain, under orf163 – a C-terminal intimin domain, under orf164 – bacterial von Willebrand's factor domain, within RBTH_07677 – LysM domains. Abbreviations include: Lg. ter. – large terminase; c.f. – putative curly fiber protein gene; pr./scaf. – protease with nested scaffold gene; h.d. – putative head decoration gene; TMP – tape measure protein; hub – homologue of T4gp27; V – homologue of P2 gpV; J – homologue of P2 gpJ.

Given numerous blast matches between 0305φ8-36 and BtI1 [1], the two genomes were subjected to a more intensive comparison of their respective gene organizations (Figure 2). The second known 0305φ8-36-related chromosomal region, BwK1, is essentially a smaller version of BtI1, so only BtI1 is graphed. We altered some of the BtI1 start sites from its GenBank entry to conform to the 0305φ8-36 annotation, and also repaired a few BtI1 frameshifts that appeared to be sequencing errors. BwK1, where present, agreed with the 0305φ8-36 annotation in these places. The graph was created by a semi-automated method for finding chains of blast matches in order and connecting them with glyphs representing the sizes of insertions or deletions (indels) between the two genomes. Decreasing shades of red indicate increasing reliance on positional information to augment blast scores. The two brightest shades of red indicate matches found by the annotation-independent, and annotation-dependent methods, respectively, as described in methods. The lightest shade of red indicates segments proposed to be homologous by means other than blast matching. Figure 2 exemplifies what we mean by genes being in the same order in both genomes.

In computing the 0305φ8-36/BtI1 genome comparison, some confusion was caused by the incidence of paralogues in both genomes. Paralogues are genes (or domains) derived from an ancient duplication and then remaining in the same genome. The existence of paralogues implies both a functional relationship between the two genes, and some degree of functional specialization to enforce retention of both of them. To help clarify the comparison between the two genomes, 0305φ8-36 paralogous domains were detected by including all 0305φ8-36 gene products in the local version of the nr library used for all Psi-Blast searches. Paralogous domains are shown in Figure 2 between the 0305φ8-36 orfs and the BtI1 track and are marked by a family designation *a*, *b, c*, etc. The paralogue track was limited to families that were close enough that the common ancestral function was plausibly phage related. Some potentially more distant relationships, for example domains sharing a fibronectin type III fold, are marked as features immediately under the orf glyphs. Paralogous domains are used below to provide insight into the evolution and/or functional assignments of numbers of genes.

The order of homologues along the genome between 0305φ8-36 and BtI1 has been retained, despite numerous insertions and deletions of genes and domains among them. Hence, the gene order has remained intact over 2 Byr of vertical descent in each of the two lineages. The revisions presumably involve horizontal gene transfer, but these have not disrupted the overall genome plan for encoding virion proteins. Even more remarkably, most functionally assigned genes conform to the most common gene order found in tailed phages [11]. Hence, the processes inferred to reconcile the vertical descent of 0305φ8-36 and BtI1 with the high incidence of horizontal transfers should apply beyond 0305φ8-36-like phages.

#### Extra structural complexity of 0305φ8-36 is encoded in 4 large modules

In the region overlapping BtI1, 0305φ8-36 has 16 more virion protein-encoding genes (27 genes replacing 11) and 13% more coding sequence [1]. It is possible that some virion protein-encoding genes of BtI1 have been excluded because the BtI1 contig ends in the indicated intein inserted in its large terminase homologue. The large modular differences between 0305φ8-36 and BtI1 consist of one substitution of 6 genes for 8 genes (orfs 165 – 170), and 3 large apparent modular insertions (orfs 119–121; orfs 126–134; orfs 152–161). These are more accurately called "indels", since they may be insertions into 0305φ8-36 or deletions during descent of BtI1.

To interpret the indels missing from BtI1 as modules requires that these genes have not been lost by random deletion in a non-functional phage relic. Random deletion can be excluded based on the absence of fragmented genes at the indel junctions, since genomes under selection for function are expected to avoid or subsequently remove defects in their frame organization [9,10]. At all of the prospective module junctions except the one in orf135, the BtI1 homology disappears at a spot between genes in both genomes. The junction in orf135 is at a domain boundary as defined by the position of a member of paralogue family *a*. Hence, the large modular differences between 0305φ8-36 and BtI1 reflect biologically selected additions or deletions of multiple virion proteins at a time.

The indels including orfs 119–121 and orfs 126–134 encode candidates for high copy number curly fiber proteins [1]. They also encode six virion proteins present in low copy number. While no homologues of these six proteins were found in outside sources, domains within gp133, gp134, and gp135 had homology to other 0305φ8-36 orfs (Figure 2, paralogue families *a*, *b*, and *c*). Paralogue family *a *appeared in six orfs (five orfs on Figure 2 and orf197 on Figure 1), and consisted of an internally repetitious sequence of about 50 residues (not shown). Paralogue families *a*, *b *and *c *are not present anywhere within BtI1 or BwK1. Some of the gene products containing family *a *or *c *are essentially composed of nothing but the paralogue domain, yet still assemble into the virion structure. So these domains are apparently able to attach to the virion by themselves, and may therefore anchor other domains with which they are fused to the virion. For example, gp154 is tentatively identified as a beta-glucosidase [1] – an activity potentially used for degrading extracellular polymer. Its fusion to paralogue domain *a *should anchor this activity to the virion, allowing the virion to clear a path to the cell surface.

#### The long tail of 0305φ8-36 is an anciently derived property

The 0305φ8-36 tape measure function has been assigned to orf146 based mainly on its correlation to tail length [1]. Blast had not found a homologue for gp146 in BtI1 or BwK1, but a gene of similar length is in the same position (Figure 2). In the original annotation of BtI1 two genes were opposite 0305φ8-36 orf146. But one gene spans the distance in BwK1 and a single frameshift would fuse the two BtI1 genes to produce the same sized gene product. Therefore, we assume that the frameshift in BtI1 is an error in the draft sequence. The positionally biased Blast search aligned only the last 60 residues between 0305φ8-36 orf146 and the presumptive BtI1/BwK1 homologue. However, the T4 tape measure (gp29) similarly diverges rapidly, becoming unrecognizable by Blast in the schizo- and exo-T4 phages (not shown), so loss of detectable sequence similarity does not dispute the assignment. We conclude that a long tail was already present in the 2.0 Byr old ancestor to 0305φ8-36.

#### Phage 0305φ8-36 has a two-gene form of the tail chaperonin

Many tailed phages have a tail chaperonin produced by a programmed translational frameshift within a pair of overlapping orfs upstream of the tape measure gene [23,24]. The prototypes are the bacteriophage λ G and T genes. Although these two sequences are not well enough conserved in most phages to be recognized by Blast, they are recognized in a broad range of phages by their position preceding the tape measure genes and their overlapping frame organization [23]. Orfs 143 and 144 are the only non-structure genes anywhere near the tape measure genes. They are one gene removed from the tape measure gene, which is an arrangement seen for some other phages [23]. Hence, Orfs 143 and 144 were examined for the chaperonin role. Although no evidence for a frameshift was found, it was noted that the C-terminal domain of gp143 was homologous to the N-terminal domain of gp144 (Figure 2, paralogue family *g*). This arrangement essentially recapitulates the relationship between λ gene products G and GT without using a frameshift.

**Figure 3:**
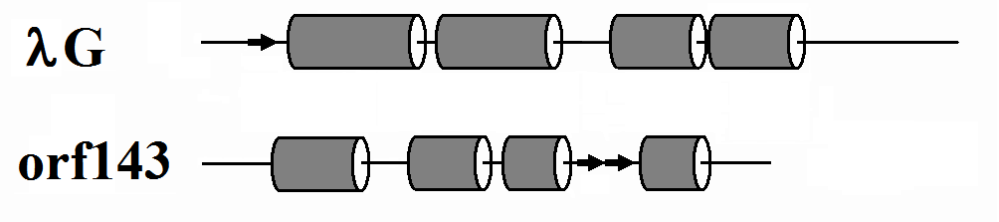
Comparison of predicted secondary structure between bacteriophage λ gpG and 0305φ8-36 gp143/gp144.

Additional evidence of homology between λ GT and 0305φ8-36 gp143/144 include the following: 1) Comparison of predicted secondary structures within λ G and the conserved portion of 0305φ8-36 orfs 143/144 reveals that both are mainly composed of four alpha helixes (Figure 3). 2) Although λ T and its homologues are of less consistent structure due to variable length, they are generally composed of additional alpha helical segments by secondary structure prediction. Correspondingly, the unique C-terminal portion of gp143 fits that description (not shown). 3) The λ GT protein is produced at only about 4% of the G product in λ [24]. Orf144 is probably also produced at low levels based on it having essentially no recognizable ribosome binding sequence (not shown). And 4) λ GT, and 0305φ8-36 orfs 143 and 144 are each in the highest 5% quantile for net negative charge. There is one discrepancy in equating gp143/gp144 to λ G/GT, which is an extra N-terminal domain on gp143 by comparison to λ gpG. But the BtI1 homologue lacks the extra domain justifying ignoring it for the more distant comparison to other phage types (Figure 2). Hence, we are confident that 0305φ8-36 gp143 and gp144 are the equivalent of the λ G/GT chaperonin system.

#### Divergence patterns in the descent of 0305φ8-36

The above observations are well precedented in comparative studies of less divergent phage genomes. These observations validate that pushing the limits of the comparative methods enables recovery of similar information in the context of a highly divergent comparison. We now apply these methods to seeking information about the 0305φ8-36 genome where there is less prior information to go on. Because the comparisons encompass so much evolutionary time, we envision observed genome rearrangements as representing an ongoing process rather than as singular events.

#### Gp142/gp209 exhibit a potential intragenomic domain transfer

Gp142 is a virion protein of unknown function. It shares a domain (Figure 2, paralogue family *f*) with orf209 – a virion protein-encoding orf also of unknown function which is an apparent moron in the right arm (Figure 1). The *f *domain is absent from the BtI1 homologue of gp142. An evolutionary scenario to do this in one recombination would require an intragenomic recombination transferring the *f *domain from an ancient version of orf209 to create an insertion in orf142. The percent identity between the family *f *paralogues is only 41%, indicating that the transfer was an ancient event. Since morons are thought to come and go frequently [8], many virion structural domains could have been acquired by this process even though the domain-donating morons are no longer present in the genome.

#### Extensive remodelling of the baseplate hub may also involve intragenomic domain transfer

**Figure 4:**
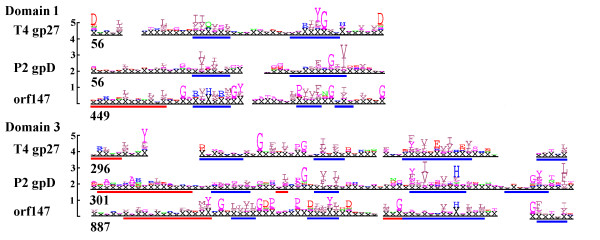
**Homology among the T4 gp 27 hub family, the P2 gpD family, and the 0305φ8-36 gp147 family**. Domain 1 and 3 refer to folding domains described for the T4 gp27 hub [26]. Sequences within each family were aligned by SAM, and converted to logos as indicated in Methods. The logo segments shown are aligned with each other as found by HHSearch [25] without assistance from secondary structure. Secondary structure was annotated subsequent to the alignment to act as a second opinion on its quality. Red and blue bars below the T4 logos represent α helixes and β strands from the crystal structure. Red and blue bars below the other logos represent secondary structure predictions.

Gp147 from 0305φ8-36 was functionally assigned as a homologue of T4 hub protein gp27 through the use of hidden Markov models (HMMs) of myoviral protein families starting with the virion proteins of bacteriophage P2 [1]. The HMM developed from P2 gpD was able to identify over 1200 homologues in phage and bacterial genomes, including one gene in nearly all known myoviral genomes and including T4 gp27 and its known homologues from T4-like phages. The HMM comparison program, HHSearch [25], found the T4 gp27 3D structure [26] within the HHpred pdb HMM library [27] using the P2 gpD HMM as the search key with E = 1 × 10^-14^, allowing a functional assignment to all members of the family. Gp147 from 0305φ8-36 was among the most divergent family members, matching in only folding domains 1 and 3 of the 4 domain structure (Figure 4). The match in domain 3 was strong enough to allow SAM to pick orf147 out of the 0305φ8-36 genome with E = 6.5 × 10^-8^. An HMM was composed from 0305φ8-36 gp147 and its BtI1 homologue and embedded in the HHpred HHM library. HHSearch picked out the gp147 model on the strength of the domain 3 match at E = 0.11. The domain 1 match was subsequently found by an HHM versus single HHM HHSearch comparison at E = 0.015. There is suitable length of sequence in gp147 to form domains 2 and 4, but the sequence is more divergent in these regions in all comparisons and these domains are not recognizable between 0305φ8-36 gp147 and its BtI1 homologue. Structurally, the two recognizable domains form a ring proximal to the end of the tail tube, whereas the two unrecognizable domains project towards the lysozyme chamber of the hub [26].

Gp147 is a much larger and more complex protein than the T4 protein. T4 gp27 organizes the assembly of the tail lysozyme and the tape measure and then the subsequent assembly of additional base plate components [26,28]. The T4 gp27 homology domain within 0305φ8-36 gp147 occupies only about a quarter of the gene product (feature marked under orf147 in Figure 2). This domain is conserved in BtI1 while there has been considerable revision of the N- and C-terminal domains attached to it. These N and C-terminal domains in 0305φ8-36 gp147 are recognized by a Pfam search as cell wall degradative domains. Gp147 has an N-terminal transglycosylase domain, and C-terminal NLP (pfam0087), and peptidase_M23 (pfam01551) domains. Both of these domains are suitable to degrade peptidoglycan, and are widely distributed in cellular lysins, phage lysins, and phage virion proteins. The BtI1 homologue has instead an N-terminal domain related to staphylococcal nuclease as annotated in the draft sequence. Further upstream, the BwK1 homologue also has an additional functionally unidentified N-terminal domain which can also be found in the BtI1 homologue if the start codon is moved upstream. The implication is that these domains occupy the position in the hub analogous to the tail lysozyme in T4, and are similarly used in the initial attack on the cell wall. The utility of the BtI1 domains is still obscure, but the 0305φ8-36 gp147 domains are clearly appropriate to help cut a hole in peptidoglycan.

Curiously, paralogues for both of the 0305φ8-36 gp147 peptidase domains are found in BtI1 just downstream of the gp147 homologue (Figure 2). Both of those BtI1 genes have the classic structure of a gram positive endolysin with C-terminal cell wall binding domains and N-terminal peptidoglycan degrading domains [29], and both are absent in phage 0305φ8-36. It is unclear if the BtI1 paralogues are truly endolysins or have been recruited to be tail lysozymes. In both cases, the BtI1 domains are not among the most similar sequences in the overall protein database to 0305φ8-36 gp147. So it is not correct to picture gp147 as directly assembled by recombination with these particular BtI1 genes. But it does indicate that these domains are of the type suitable to have been imported as endolysins, and then reutilised by intragenomic recombinations to decorate virion proteins. Although it is not obvious why the BtI1 hub protein carries a staphylococcal nuclease domain, that domain is also known to have been imported into several phages as a stand-alone gene (see Pfam00565). We suspect that these domains were all intragenomic transfers from stand-alone genes, whether or not the stand-alone gene is still present in the viral genome.

#### Additional baseplate/fiber genes maintain order in spite of extensive recombinational revision

Both by the most common gene order [11] and by elimination, genes downstream of orf151 should encode additional baseplate components and/or fibers or other appendages. Blast matches in this area are typically to widely used folding domains, most typically fibronectin type III folds (Fn3) (gps 163, 165, 166, 167). These could be binding domains for viral assembly or for host or environment interaction, but the Blast matches do not extend to parts of the matched proteins that would reveal specific functions. There are also a significant number of coiled coil regions detected (gps 163, 164, 168, 169, 170, 171, 172, 173, 174, 175), which are typically used in protein-protein interaction. The region covered by orfs 162 to 164 is particularly chaotic in its relationship to BtI1 (Figure 2), but remarkably the 5 blast matches to BtI1 remaining in the area fall in a consistent order.

The loss of similarity in between the blast matches in the orf 162–164 region has more to do with domain substitution than with divergence beyond recognition. This is apparent from the recognized folding domains marked as features in Figure 2. The central portion of gp164 contains a bacterial von Willebrand factor, type A domain [30,31] that would have been recognized in the BtI1 and BwK1 homologues if present. Hence, the central part of this gene has been swapped for an alternative domain between 0305φ8-36 and BtI1/BwK1. Similarly, 0305φ8-36 gp163 contains a C-terminal intimin domain (related to a bacterial adhesion protein domain pdb 1F00 [32], and the BtI1 gene contains a Fn3 domain not found in orf163. In the N-terminal portion of orf163 there is an array of four Fn3 domains not found in the paired BtI1 gene, and the paired BtI1 gene has two LysM (Pfam01476, peptidoglycan degrading) domains not found in orf163. It would take numerous recombination events to explain the restructuring of this region between 0305φ8-36 and BtI1. It therefore qualifies as a hyperplastic region of the type described for T4-like phages [16]. Hyperplastic structure gene regions tend to involve the phage proteins that actually recognize the host. Both by this criterion and in consideration of the kinds of domains in this area, orfs162–164 would appear to be excellent candidates for a major host recognition determinant of phage 0305φ8-36.

#### Organization of the right arm

The right arm lacks any sequence of genes to which it can be compared. There are, however, internal patterns of gene organization.

#### The right arm differs from the left in content of noncoding sequence

Also shown in Figure 1 (above the orfs) is the distribution of noncoding segments of sufficient size to encompass a promoter. There are noticeably more non-coding spaces in the right arm in spite of the fact that we were equally thorough in trying to fill such spaces with small orfs in both arms. Typically phage genes are tightly packed and often overlap [13,33]. When annotating a new phage genome, there are frequently arbitrary decisions to be made as to whether there is a small orf or a noncoding region between the larger orfs. In the 0305φ8-36 left arm, both by mass spectrometry survey [1] and the conservation of frames in BtI1 (Figure 2) demonstrate that the small orfs usually are real genes. The conclusion of tight packing, thus justified, implies ongoing selection for compaction. A basic model for compaction selection is that the phage acquires new genes until it suffers a negative selection penalty for the size of its genome, and then it removes low value segments of DNA to relieve the penalty. Presumably low value DNA on either arm would be susceptible to removal. Therefore, we assume that the distribution of noncoding DNA on the right arm represents a distribution of noncoding functions. In particular, we assume that noncoding segments just big enough to hold a promoter usually do have a promoter, and that the distribution of such spaces gives a rough impression of the organization of polycistronic transcripts.

#### The right arm contains a putative novel RNA polymerase gene

**Figure 5:**
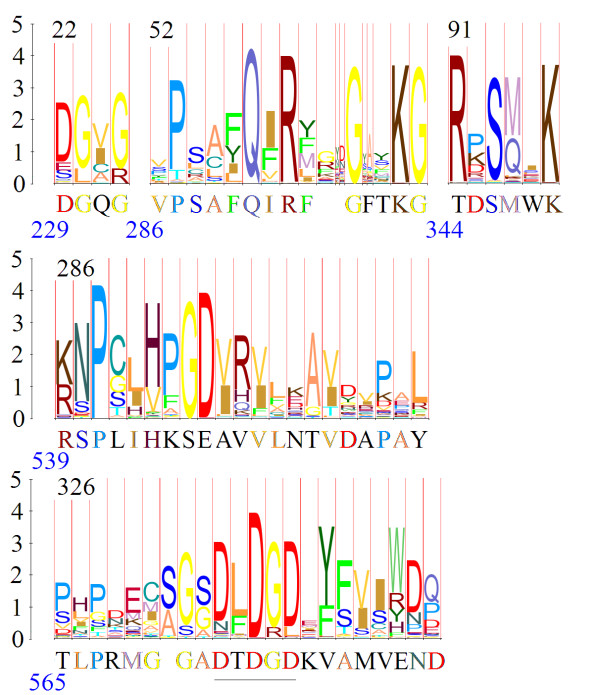
**Alignment of 0305φ8-36 orf99 to diagnostic motifs of the RNA-dependent RNA polymerase family Pfam05183**. The motifs [34] are represented by segments of the sequence logo obtained from Pfam. The orf99 sequences aligned according to SAM.

A potential factor in the transcriptional organization of 0305φ8-36 is that orf99 appears to be a phage-encoded RNA polymerase. This gene was initially found as a weak Blast match to a portion of eucaryotic RNA-directed RNA polymerases involved in amplifying RNA during an RNAi response (Pfam05183). We expanded the Pfam domain model into a complete sequence alignment and HMM model using SAM. SAM then detected 0305φ8-36 orf99 with E = 10^-100 ^and aligned it from end to end. Segments of the Pfam sequence logo described as definitive of this family [34] are shown in Figure 5 with the gp99 sequence aligned according to SAM. The family has been characterized [34] as having no detectable sequence similarity to virus-encoded RNA-directed RNA polymerases or any DNA-directed RNA polymerases. However, a role for an RNA-directed RNA polymerase in 0305φ8-36 would require it to be involved in some unprecedented process for a DNA phage. Alternatively, we tentatively assume that gp99 is a DNA-directed RNA polymerase, possibly representing the function of the ancestor of this polymerase family. Other than the obvious potential for involvement in gene expression, there is also the possibility that the polymerase is involved in some aspect of injection. However, the precedent for RNA polymerase-mediated injection is that it would probably be too slow to be used exclusively on a genome of this length [35].

**Figure 6:**
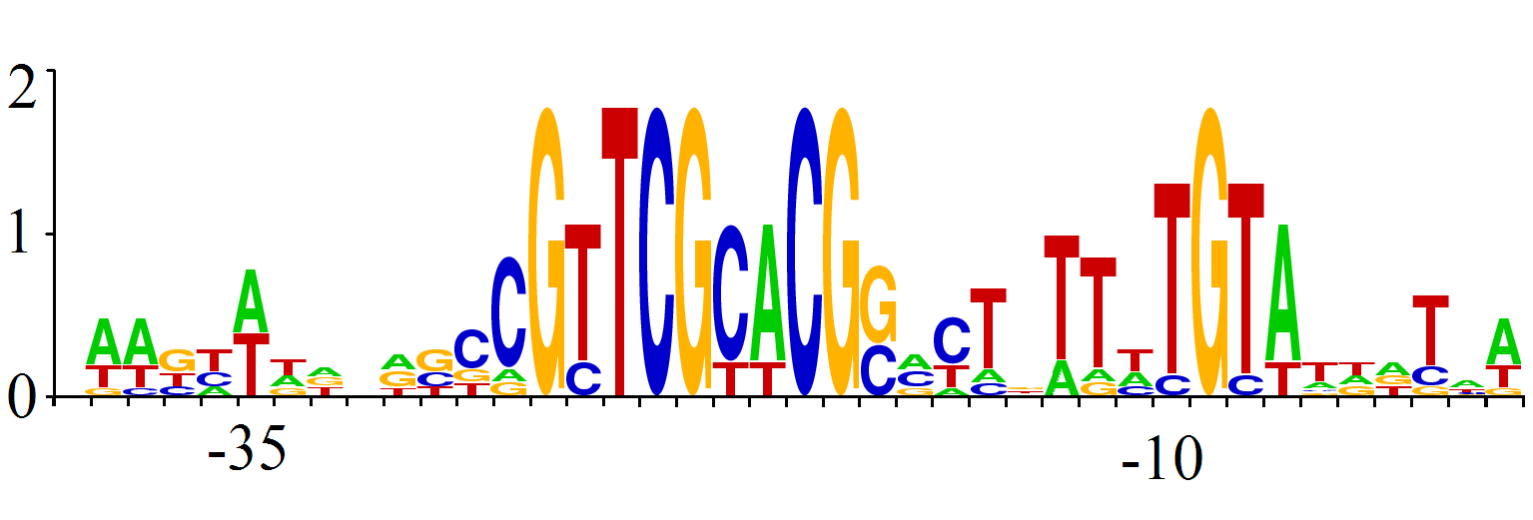
**DNA sequence logo representing 14 candidates for 0305φ8-36-specific promoters**. The corresponding 14 noncoding segments are indicated in cyan in Figure 2.

We asked if there was either a novel promoter motif, such as used by T7 RNA polymerase [36], or recognizable TATA and -35 boxes in the spaces inferred to hold promoters. One class of promoter candidates having substantial self-similarity over 21 bp is described by the sequence logo in Figure 6. Ten of these were found by inspection, and then a SAM HMM model constructed from these ten found an additional four. None were found in the *B. thuringiensis israelensis *genome. These phage-specific promoters candidates are marked on Figure 1 (cyan noncoding bars). They are appropriately distributed to be a middle expression promoter. The proposition that these are targets of the encoded polymerase is supported by the lack of recognized sigma factors encoded in the 0305φ8-36 genome. However, the possibility that host polymerase is somehow directed to these promoters can not be excluded at this time.

#### Apparent operon sizes may reveal early, middle, and late transcript organization

The orfs between 202 and 208 kb are all small, each apparently on a monocistronic transcript (Figure 1). A precedent for this organization appears in the 11.5 kb SPO1 host takeover region [37]. One theoretical explanation for this frame organization would be that these gene products are selected for rapid synthesis after DNA injection. So, to achieve rapid expression, they consist of short frames on short transcripts. Transcription elongation occurs at 40–80 nt/s [38]. Hence, the advantage of keeping these genes separate would amount to a few seconds. If seconds are important, then the right end must be first into the cell. The putative RNA polymerase gene is also on a monocistronic transcript, consistent with a requirement for rapid expression.

Downstream from the host-takeover region, monocistronic frames are phased out in 0305φ8-36 between orf91 and orf80 with the inclusion of two apparent three-gene operons of about 1.5 total kb each. The virion protein-encoding gene, orf81, is organized like a moron with a downstream transcription terminator that would block read through from its own promoter and upstream promoters. Downstream of orf81, most operons are longer with several up to about 6 kb and encoding up to seven genes. However, these apparent transcripts are still only about half the size of those in the structure gene region. This results in 30 potential promoters on the remainder of the right arm. We propose that the abundance of promoters in this part of the right arm is to limit the delay in expression of these genes to the few minutes it takes to transcribe 6 kb. In essence the proposal is that gene organization throughout the genome, rather than just at the leading end, is influenced by time of injection. However, there is a complication introduced by the intermixing of the 21 bp promoter motif (Figure 6) with apparent promoters not containing this motif. This pattern implies that transcription control of the nonstructure genes is transferred from one polymerase complex to another at some point during infection, and that actual transcript sizes may therefore fluctuate with time after infection.

#### There is limited functional clustering within the right arm

Elsewhere within the right arm only limited functional clustering is seen. Co-transcribed orfs 16 and 15 are interesting in that both seem likely to modulate host functions, but are not clustered within the host takeover region. Orf16 encodes a mazG homologue thought to degrade ppGpp and hence preclude inhibition of translation during a stringent response [39], and orf15 encodes a serine/threonine phosphatase. Proteins that work in a complex appear to often be encoded next to each other and on the same transcript. Examples are: 1) α and β ribonucleotide reductase and the associated flavodoxin, 2) α and β DNA polymerase III, and 3) the two subunits of the MoxR-type metalloprotein chaperonin [40]. Hence, the main discernable levels of organization of the non-structure genes consist of the clustering of the presumed host takeover genes, and after that the clustering of genes whose products directly interact. Even this limited clustering aids in functional assignment of the orfs. For example, the flavodoxin almost certainly is the electron carrier for the ribonucleotide reductase. Also, while orf66 is identifiable as a MoxR subunit by sequence similarity, close linkage to orf66 is a key element of the identification of orf63 as the second MoxR subunit [40].

#### Encoded replicative functions suggest fusion of two replicative systems

Phage 0305φ8-36 carries two primase genes, one in the left arm, and one in the right arm. The primase encoded on the left arm, orf181, belongs to a family normally found in *Archae *and eucaryotes (Pfam01896). This primase is found in bacteriophages and plasmids and is capable of functioning together with a variety of replicative helicases including DnaB [41]. There is an adjacent DnaB locus, orf182. The archaeal-eucaryotic primase is usually found encoded adjacent to its helicase, so we suspect that gp181 and gp182 collaborate to form a replicative complex. The primase encoded on the right arm, by orf236, belongs to the dnaG family. Replication in eubacteria is normally supported by collaboration of a dnaG primase and a dnaB helicase. The 0305φ8-36 dnaG primase is missing the domain usually used to associate with a dnaB helicase. It is unclear whether the 0305φ8-36 dnaG primase interacts with a different helicase, or perhaps associates with the dnaB helicase using another gene product as an adapter. The hypothesis that the 0305φ8-36 genome plan was formed by the fusion of separate left and right arm ancestors would provide a natural explanation for the distribution of these replication genes.

## Discussion

### The genome plan

*Bacillus thuringiensis *phage 0305φ8-36 exhibits many unusual features, including a long genome, a high degree of structural complexity as measured by total length of virion protein-encoding sequence, and a proteome that is highly divergent from known bacteria or bacteriophages [1]. We have found the left arm to be roughly 2.0 – 2.5 Byr diverged from the closest relative – a segment of cellular chromosome called BtI1. Remarkably, the genes that are homologous between 0305φ8-36 and BtI1 are still arranged in the same order. The order persists despite the region having been heavily affected by exchanges of units ranging from multigene modules to intragene domains. The left arm is estimated to be 3 Byr or more diverged from other myoviruses. The right arm genes are similarly estimated to be ca. 3 Byr diverged from their closest homologues. We have not determined if those homologues are prophage genes or ordinary cellular genes, although they were not found anywhere clustered together as might be expected for a similarly organized prophage. Hence, the 3 Bya mark may represent the original recruitment of this collection of cellular genes to viral function.

The left and right arms of 0305φ8-36 have properties suggesting that two different viral ancestors were fused to make the 0305φ8-36 genome plan at some ancient time (see results). It is not unusual to see patterns of homology suggesting a bulk exchange of the non-structure genes (*e.g*. VpV262 versus SIO1 [19]). The 0305φ8-36 genome plan is, however, unusual in retaining at least a portion of the replication systems of both postulated ancestors, one in the left arm and one in the right arm. This implies that the fusion was not accomplished in one step by an unequal crossover between the structure region of one phage and the non-structure region of another. Instead there was a more extensive inclusion of at least the nonstructure genes from both ancestral phages followed by elimination of duplicated functions by intragenomic recombinations. This is a large scale application of the two step process proposed to restore close packed frame organization after horizontal transfers [9,11]. Intragenomic recombination can occur rapidly because there are many opportunities during every phage infection. The multi-step process leaves the more exacting streamlining recombinations to secondary intragenomic recombinations. The rate limiting intergenomic (horizontal) step can then include a wider range of imprecise recombinations than apparent from the final outcome, thus explaining a high frequency of successful transfers.

Fusions that involved duplication and reassortment of multiple genes may have created the genome plans of other phages. But if some duplicated functions are not retained, it would be hard to distinguish this complex scenario from a one step modular exchange. The division of labor that favored the retention of two primases in 0305φ8-36 is not clear. Assuming that phage 0305φ8-36 starts replication from multiple origins of replication, as in T4 [13], there may be special requirements of left arm origins not well serviced by right arm replication genes. A candidate for a left arm-specialized process would be the generation of the terminal repeat. Assuming that the primary replication process produces a concatemer, the left end repeat must be synthesized in a separate step in coordination with packaging. Since the packaging apparatus is encoded by the left arm, there may also be coadapted replicative functions retained from the left arm ancestor.

### Vertical descent and hyperplastic regions

The relative isolation of the phage 0305φ8-36 genome from horizontal exchanges with phages of other known groups mirrors the findings of recent studies of the T4 superfamily [15,16]. These studies found the genomes of T4-like phages to have core genomic regions exhibiting clean vertical descent. Regions interspersed with these core regions exhibited more frequent horizontal exchanges, and were termed "hyperplastic". The main hyperplastic structure region of the T4-like genomes encoded the distal portions of the tail fiber. Phage 0305φ8-36 has a hyperplastic region encompassing virion protein-encoding orfs 162–164, based on several horizontal exchanges marked by the presence or absence of recognizable folding domains in the 0305φ8-36/BtI1 comparison.

A key problem in analyzing the rate at which recombination reorganizes a genome is that an observed recombination junction may be just the last step in a history of multiple exchanges. The total number of exchanges affecting the comparison of 0305φ8-36 and BtI1 in the hyperplastic region can be estimated if a similar exchange rate occurred as for the T4 family. There appear to be at least 23 horizontal exchanges mapped in a collection of T4-like tail fiber genes out to vibriophage KVP40 [16]. The tree relating these phages has been described [15]. Applying a 0.8 Bya time to the *Vibrio *split from enterobacteria [22] produces a sum of branch lengths of about 2.8 Byr for the T4 family tree. So the T4 lineage experienced about eight horizontal exchanges per Byr in this hyperplastic region. This count only includes exchanges that produce a noticeable incongruency in the T4 tree. There are presumably even more exchanges of a more subtle kind. Phage 0305φ8-36 and BtI1 are separated by 4 – 5 Byr (sum of 0305φ8-36 and BtI1 branches), so the hyperplasticity observed between them in orf162–164 is projected to result from 30–40 substantially reorganizing horizontal exchanges. Yet even in the hyperplastic region the domains that match by Blast remain in the same order.

### Seeking vertical descent in divergent genomes

These observations raise the question of what keeps the genes in order. Conservative selection operating on the clustering of functions has been proposed, in particular on the genes that assemble the virion [15]. Conservative selection brought about by the need for coordinating gene expression is under exploration in the T7 system [42,43]. However, those aspects of organization could be satisfied by more than one gene order. The main factor in keeping genes in order is presumably because that is the normal outcome of phage replication. Even in the hyperplastic region, assuming one generation per day [44], the same time period that produced 30–40 horizontal exchanges will have encompassed ~10^12 ^generations characterized by organizationally conservative vertical descent. This is not to deny that horizontal exchanges can have disproportionate biological consequences, and can make conceptualization of the evolutionary history difficult [9]. However, the 0305φ8-36/BtI1 comparison shows that there can be extensive conserved gene order beyond the threshold of simple inspection even for highly diverged phage genomes.

To conduct a thorough domain by domain comparison of two divergent phage genomes from standard blast listings is taxing, as is generating a visual depiction of the results. Figure 2 was modified from the output of an algorithm designed to speed up the process. The algorithm incorporates three lessons derived from algorithms like Blast designed to effectively recognize divergent amino acid sequences. (1) The significance of a match must be evaluated in the context of whether it belongs to a sequence of matches. (2) Some provision must be made to allow for insertions and deletions. And (3) collating and graphing the results is a job best done by a computer. What emerges is an extended pattern of similarity representing the vertical descent of the core structural determinants of the 0305φ8-36 virion. Also visualized are patterns of change, which carry information about the modes of evolution affecting the phage.

### Intragenomic exchange

Several patterns of domain reuse cited in the results can be interpreted in terms of a process by which intragenomic exchange modifies the vertically conserved genes. They are 1) the reuse of paralogue family *a *to anchor multiple proteins of unknown function to the phage, 2) the fusion of virion proteins with cell wall binding, and peptidoglycan degrading domains postulated to come from previously acquired lysins, and 3) the fusion of structural anchoring domains with a putative stand-alone capsular polymer degrading enzyme. The proposal is that there is a class of domains frequently decorating virion proteins, but first arriving in the genome as morons encoding stand-alone proteins. This would have the accelerating effect described above for domains that can function in stand-alone proteins, and bias the evolutionary process to use these particular domains more often than others for elaboration of modified virion structure.

Discriminating domains that tend to transfer laterally from those that tend to descend vertically is an aid in recognizing the relationship between genomes. An example is illustrated in the analysis of orf147 and RBTH_07687 in Figure 2. Aligning the peptidoglycan cleaving domains would force the T4 gp27 hub homologous domains out of alignment. The software reports both alignments. The pictured alignment emphasizes the domain coadapted to assemble with the other virion proteins and hence arriving by vertical descent. In many cases, the information to identify the assembly domain would be absent. But domains that attack peptidoglycan are now well documented in Pfam, so the assembly domain may be inferred by elimination.

### Vertical descent and the right arm

The algorithm to detect and graph genes in order was also used to exclude relationship by vertical descent of the right arm to other known genomes. We have postulated that the right arm is derived from a separate and ancient ancestral virus. The right arm has interesting features we would like to subject to comparative analysis, such as the presence of the RNA polymerase gene, the degree of mosaicism, or the way the density of promoter-sized noncoding regions suggests a coordination of transcriptional control with injection (see results). So for each best blast match we computed a display like that in Figure 2 between 0305φ8-36 and the chromosome matched. No clustering of related genes in other chromosomes has thus far been found. However, the method is relatively expedient in testing new candidate chromosomes to find one that would enable asking these questions.

### Modular exchange and structural complexity of 0305φ8-36

Finally, the method of Figure 2 defines four multigene modules of genes encoding virion proteins that differ between 0305φ8-36 and BtI1. Since the total length of 0305φ8-36 structure genes is twice that of T4 [1], these modules appear to represent additions to the basic myoviral virion structural plan. Possible biological roles of the additional structural genes in 0305φ8-36 have been discussed [1]. The following possible implications of multigene modules have been reviewed [8]: 1) that the genes have been acquired at once, and/or 2) that the genes collaborate on some function. These two interpretations tend to be tied together in the case where a new function is acquired by horizontal transfer. This is because the function can only transfer if all necessary genes are transferred, and functionally unrelated genes in the transferred module will tend to be subsequently lost. On the other hand, separation into different modules does not necessarily imply that the genes are functionally independent. Instead all of the genes may have initially been organized in one module. Then a second version of one or more of the genes may have been acquired at a different locus, followed by the loss of one each of the duplicated genes.

Questions that may be addressed by the modular structure of 0305φ8-36 include: 1) whether the extra virion proteins have been added in several independent assemblies, and 2) whether this was done early in the 0305φ8-36/BtI1 lineage or later in 0305φ8-36 alone. As argued above, the presence of four extra 0305φ8-36 modules does not necessarily imply the addition of four separate structural assemblies. There is an indication of functional links among these modules in the repeated paralogous domains (paralogue families a, b, c) distributed in three of the modules. As described in the results, the repeated domains may represent a virion anchorage system used in common by the structures encoded in these modules. This would then further suggest the invention of both a novel anchorage system and its use to elaborate additional structure in 0305φ8-36 since the 0305φ8-36/BtI1 split. Consistent with this theory, 0305φ8-36 contains 70% more virion protein-encoding gene sequence than the set of structure genes homologous with BtI1. However, much of the difference between 0305φ8-36 and BtI1 is compensated by a 14 kb module of BtI1 genes substituted for 0305φ8-36 orfs 166–170 (Figure 2). If these BtI1 genes also encode virion proteins, then BtI1 encodes nearly as structurally complex a virion as 0305φ8-36, except with a different component of novel virion proteins. So the possibility exists that the 0305φ8-36/BtI1 lineage became committed very early to a highly complex virion structure but maintains this commitment with several alternative sets of structural assemblies. Since the curly fibers are apparently part of the extra structural component of this lineage, further insight into this unusual system may be obtained by identifying and sequencing other phages carrying large curly fibers.

## Methods

Figure 1 was the output of graphics display program Gbrowse [20] dynamically linked to a locally maintained annotation database for this phage. Figure 2 was modified from the output of a program, b36chain, written in the course of this study to add comparative genomic data to the Gbrowse display. Two different methods were employed for incorporating positional information to highly divergent homologues as follows. Program b36chain conducts the equivalent of a Tblastn search between the genome under analysis and a genome selected on the basis of one or more significant matches from a standard database search. There is an inherent improvement in sensitivity because the E values used to reject chance matches will have been recalculated based on the size of the subject genome rather than on the size of the entire nr database. The results are collated by position on both genomes. The E-values of weak matches were then further improved by 3/219 (window length/genome length) if they fell within a 3 kb window around the same established match on both genomes. Matches thus elevated beyond a threshold of significance were then treated as established matches for evaluation of the next 3 kb interval. The program then produced a gff file which directs Gbrowse to add a track with a chain of glyphs representing matches found in the same orientation and order in both genomes and within 3 kb of the same spacing. The program inserts connector glyphs representing insertions or deletions between the matches and scales these to the amount of DNA gained or lost. In the case of conflicting geometry, multiple chains are drawn representing the alternative alignments of the matches. An additional track is also provided reporting the coordinates of each match on the subject and target genomes before positional filtering (not shown). That track defined how the coordinates of the subject genome must be folded to align with 0305φ8-36 coordinates for use in the second method described below. The unfiltered track shows in this case that there are not plausible divergent relationships other than the indicated matches found in order. The image produced was hand edited to resolve alternative alignments due to repeated sequences in conjunction with creation of a paralogous domain track. This method, represented by the darkest shade of red in Figure 2 is annotation independent. It is nearly completely automated and does not require prior prediction of frames on either genome.

The second method to incorporate positional information made use of the annotation for both genomes. Some improvements in BtI1 start codon positions and some additional unannotated BtI1 genes were also incorporated. Annotated frames in each genome that were aligned but not matched by the annotation independent method were subjected to a BlastP search by the "Blast 2 sequences" service at NCBI. The E value for acceptance was arbitrarily increased to a maximum value that still excluded random matches appearing off diagonal on the dot plot of the output. On-diagonal matches were then included in Figure 2 at the second level of red. This method is called the "annotation-dependent" method in the text, and is not automated at this time.

Secondary structure prediction and HMM modelling with SAM were as described [1]. Charge distribution was calculated at the Statistical Analysis of Protein Sequence web server [45,46]. Figure 4 was derived from logos created by the SAM makelogo utility after incorporation of prior information by the w0.5 utility [47]. Figure 5 was derived from a sequence logo created at Pfam [48]. Figure 6 was produced at the WebLogo web server [49,50].

## Competing interests

The author(s) declare that they have no competing interests.

## Authors' contributions

SCH designed the study, performed informatic analysis with respect to genomic organization, and wrote the paper. JAT performed informatics analysis with respect to functional gene assignment and wrote portions of the paper. PS participated in the design and coordination of the study and helped draft the manuscript.
